# Day-to-day pattern of work and leisure time physical behaviours: are low socioeconomic status adults couch potatoes or work warriors?

**DOI:** 10.1186/s12889-021-11409-0

**Published:** 2021-07-07

**Authors:** Charlotte Lund Rasmussen, Dorothea Dumuid, Karel Hron, Nidhi Gupta, Marie Birk Jørgensen, Kirsten Nabe-Nielsen, Andreas Holtermann

**Affiliations:** 1 National Research Centre for the Working Environment, Copenhagen, Denmark; 2 Allied Health & Human Performance, Alliance for Research in Exercise, Nutrition, and Activity (ARENA), University of South Australia, Adelaide, South Australia Australia; 3 Department of Mathematical Analysis and Applications of Mathematics, Palacký University, Olomouc, Czech Republic; 4 Occupational Health and Safety, Municipality of Copenhagen, Copenhagen, Denmark; 5 Section of Social Medicine, Department of Public Health, University of Copenhagen, Copenhagen, Denmark; 6 Department of Sports Science and Clinical Biomechanics, University of Southern Denmark, Odense, Denmark

**Keywords:** Physical activity, Sedentary time, Compositional data analysis, Time-use, Socioeconomic inequality, Accelerometer data

## Abstract

**Background:**

Most studies on day-to-day patterns of physical behaviours (i.e. physical activities and sedentary behaviour) are based on adults with high socioeconomic status (SES) and without differentiating between work and leisure time. Thus, we aimed to characterise the day-to-day leisure time physical behaviours patterns among low SES adults and investigate the influence of work physical behaviours.

**Methods:**

This cross-sectional study included 963 adults from low SES occupations (e.g. manufacturing, cleaning and transportation). The participants wore accelerometers for 1–7 days to measure physical behaviours during work and leisure time, expressed as time-use compositions consisting of time spent sedentary, standing or being active (walking, running, stair climbing, or cycling). Compositional multivariate multilevel models were used to regress daily leisure time-use composition against work time-use compositions. Interaction between weekday and (1) type of day, (i.e., work/non-work) and (2) the work time-use composition were tested. Compositional isotemporal substitution was used to interpret the estimates from the models.

**Results:**

Each weekday, workers consistently spent most leisure time being sedentary and most work time standing. Leisure time physical behaviours were associated with type of day (*p* < 0.005, more sedentary on workdays vs. non-workdays), weekday (*p* < 0.005, more sedentary on Friday, Saturday and Sunday), standing work (*p* < 0.005, more sedentary and less standing and active leisure time on Sunday), and active work (*p* < 0.005, less sedentary and more standing and active leisure time on Sunday). Sedentary leisure time increased by 18 min, while standing and active leisure time decreased by 11 and 7 min, respectively, when 30 min were reallocated to standing at work on Sunday. Conversely, sedentary leisure time decreased by 25 min, and standing and active leisure time increased by 15 and 10 min, respectively, when 30 min were reallocated to active time at work on Sunday.

**Conclusions:**

While low SES adults’ leisure time was mostly sedentary, their work time was predominantly standing. Work physical behaviours differently influenced day-to-day leisure time behaviours. Thus, public health initiatives aiming to change leisure time behaviours among low SES adults should consider the influence of work physical behaviours.

**Supplementary Information:**

The online version contains supplementary material available at 10.1186/s12889-021-11409-0.

## Background

Leisure time physical behaviours (i.e. physical activities (PA) and sedentary behaviour) are well-known determinants of non-communicable diseases and mortality [1–4]. Worldwide efforts are made to increase PA [5, 6]. Nevertheless, current PA promoting interventions and policies seem to fail in reaching those in most need of health-enhancing leisure time PA: individuals of lower socioeconomic status (SES). Current data on global leisure time PA levels show a persistent, steadily widening SES gap, favouring high SES groups [7–9]. Clearly, there is an urgent need to strengthen PA promoting strategies to narrow this gap and thus, decrease inequalities in health.

One strategy to increase PA levels among low SES adults could be to recommend health enhancing physical activities on non-workdays days (during the week or weekends) with more energy and free time available than on workdays. This pattern of higher PA, e.g. during non-working weekends, than on workdays has been found among high SES adults (termed “weekend warriors”) [10–12]. An opposite pattern, with more PA on weekdays than during the weekend has been found among low SES adults [13, 14]. However, none of the previous studies differentiated between work and leisure PA. As low SES adults are often in blue collar positions with high levels of PA as part of their job [15, 16], occupational PA is likely the driving factor for their high accumulation of PA on workdays.

The few studies assessing determinants of physical behaviour patterns over the week have primarily focused on individual factors, such as age [17], income [11] and education [13]. However, at best, individual-level factors explain 20–40% of the variance in PA levels [18]. Thus, research and policies on physical behaviours has increasingly adopted a broader approach which also considers environmental determinants, such as work factors [19, 20]. Understanding how work factors could affect daily leisure time physical behaviours is considered particularly important among low SES groups, as such factors are modifiable and amenable to change with interventions [21, 22]. Nevertheless, to our knowledge, no study has investigated how work factors, such as work physical behaviours, influence day-to-day leisure time physical behaviours. Such insight into domain-specific physical behaviours over the week and potentially modifiable determinants is needed to inform approaches aiming to increasing health-enhancing leisure time PA. As highlighted by the World Health Organization (WHO), this knowledge on domain-specific physical behaviour patterns is lacking, yet, urgently needed among less privileged adults [23].

When investigating the relationship between work and leisure time physical behaviours, it is essential to consider the potential co-dependency between these behaviours. That is, increasing time spent in one behaviour may take away time from another behaviour. Consequently, work and leisure time physical behaviours are likely to be co-dependent and collinear. These type of data are known as compositional data and they convey *relative* rather than *absolute* information [24]. Accordingly, assessment of patterns and associations between domain-specific physical behaviours can be made in terms of relative information [25, 26]. This is done by using compositional data analysis (CoDA), which is based on a log-ratio methodology. To our knowledge, no study has assessed the day-to-day pattern of domain specific physical behaviours while taking the co-dependency between time-use behaviours into account.

The aim of this study was to characterize the day-to-day pattern of work and leisure time physical behaviours among low SES adults. Moreover, we investigated the influence of day of the week and its interaction with type of day (i.e. workday vs. non-workday) and work physical behaviours on day-to-day leisure time physical behaviours using a CoDA approach.

## Methods

This study used baseline data from two Danish studies with identical data procedure and collection: the Danish PHysical ACTivity cohort with Objective measurements (DPhacto) [27] and the New Method for Objective Measurements of Physical Activity in Daily Living (NOMAD) study [28]. The study population consisted of low SES workers recruited from Danish workplaces within cleaning, transportation, manufacturing, construction, road maintenance, garbage disposal, assembly, mobile plant operator, and health care. Workers were eligible if they were employed in one of the mentioned sectors for at least 20 h/week; between 18 and 65 years old; and had given voluntary consent to participate. Workers were excluded if they had band-aid allergy, fever on the date of data collection or were pregnant.

### Data collection

Details of data collection and related instruments used for the DPhacto and NOMAD studies have been described previously [27, 28]. In short, data collection included questionnaires, health checks, and accelerometer-based measurements. Workers eligible for participation were invited to complete a questionnaire and to participate in a health check, which consisted of anthropometric measurements and a physical health examination. Moreover, participants were asked to wear accelerometers for a minimum of two consecutive workdays and to complete a daily diary reporting time at work; time going to bed at night and getting up in the morning; and non-wear time.

### Accelerometer measurements of physical Behaviours

Physical behaviours at work and leisure time was assessed using data from one tri-axial ActiGraph GT3X+ accelerometer (Actigraph, Pensacola, FL, USA). The accelerometer was placed on the right thigh using double-sided adhesive tape (3 M, Hair-Set, St. Paul, MN, USA) and Fixomull (Fixomull BSN medical GmbH, Hamburg, Germany). Accelerometer data were downloaded using the Actilife Software version 5.5 (Actigraph, Pensacola, FL, USA) [29] and analysed using the custom-made MATLAB program Acti4 (The National Research Centre for the Working Environment, Copenhagen, Denmark) [30]. In brief, the Acti4 program derive instantaneous average parameters (i.e. mean and standard deviation of acceleration, inclination) from thigh-worn accelerometer signal using overlapping 2-s intervals. Based on these parameters and a rule-based decision tree, physical behaviours (i.e., cycling, stair climbing, running, walking, standing, sitting and lying) were classified [19]. The Acti4 program has been shown to separate physical behaviours with high sensitivity and specificity under both semi-standardized [30] and non-standardized conditions [31].

Day of the week, daily work hours, leisure time and time-in-bed were defined from the participants’ daily diary. Specifically, leisure time was defined as waking time not at work. Non-workdays were defined as days where the participants had not reported a working period. Only workers having at least 1 day of valid accelerometer measurements of both work and leisure time periods were included in this study. A valid day was defined as having at least 4 h of accelerometer-derived work and leisure time or what corresponded to at least 75% of the individual’s average work and leisure time. To decrease the risk of reverse causality between work periods and leisure time, we only considered leisure time following work. Moreover, time-in-bed was not considered in this study.

**Fig. 1:**
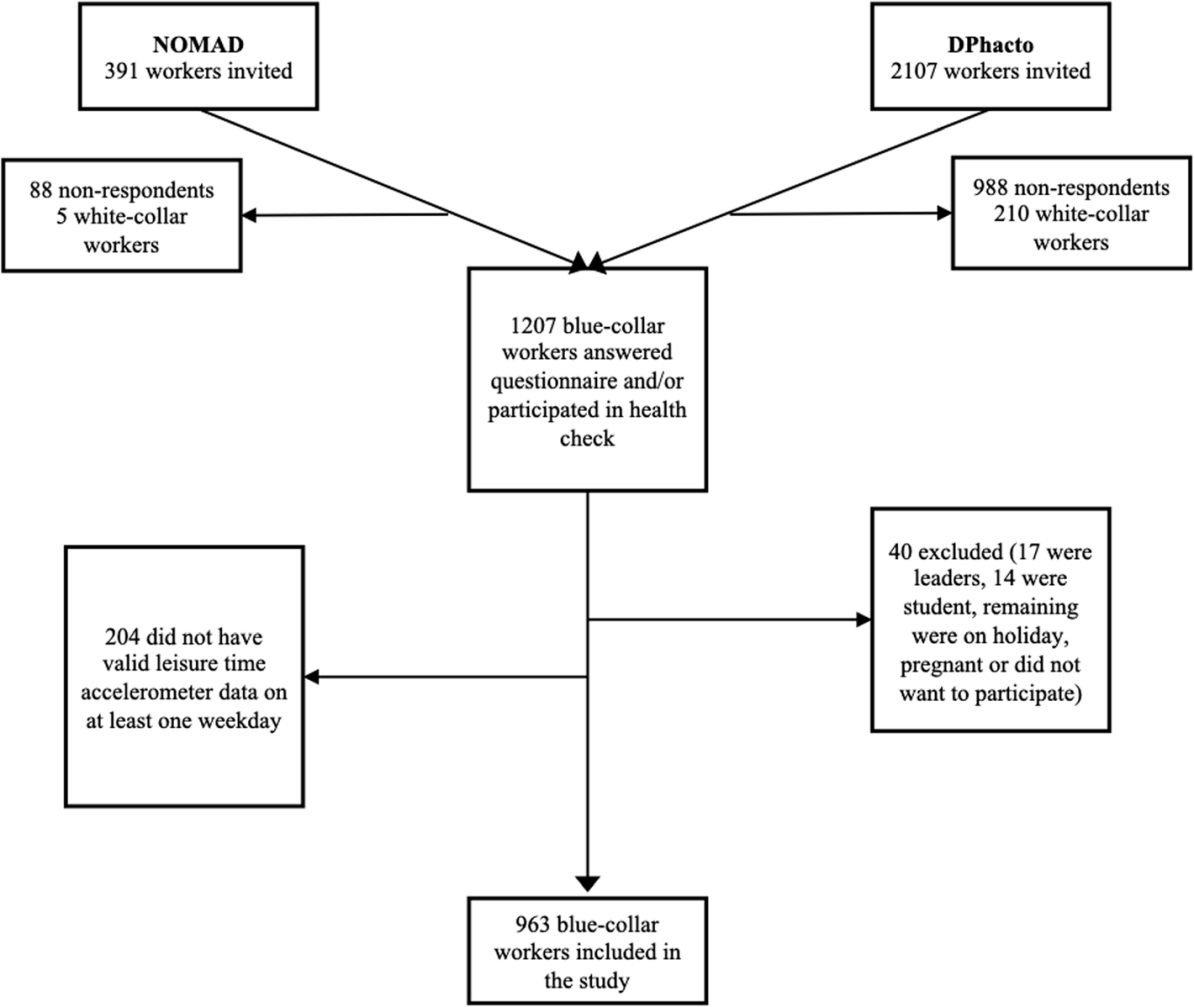
Flow chart of the study population

Figure 1 shows the flowchart of the study population. A total of 1207 eligible workers answered the questionnaire and/or participated in the physical health check. Of these workers, 40 were excluded as they were department leaders or students, on holiday, pregnant or because they did not want to participate. A total of 204 workers were excluded from the study as they did not valid have leisure time accelerometer measurements on at least one weekday. Therefore, a total of 963 workers were included in the study.

### Covariates

Sex and age were determined from each worker’s unique Danish civil registration number. BMI (Body Mass Index) was calculated as weight (kg) divided by height (m) squared (kg/m^2^). Information on smoking-status was obtained by the question: “Do you smoke?” with four response categories: daily smoking, occasionally smoking, formerly smoked, and never smoked. The variable was dichotomized into smokers and non-smokers (including former smokers). Information on shift work was obtained using the question: “At what time(s) of the day do you usually work in your main occupation?” with three response categories: fixed day work, night/varying work hours with night, and other. The variable was dichotomized into workers with and without fixed day work. Work duration was calculated as the log of total accelerometer-derived work time [32].

### Statistical analysis

#### Compositional descriptive

Time use of daily work and leisure time behaviours was treated as two compositions of activities performed within a 24-h day. Work and leisure time were defined as a 3-part composition, both consisting of time spent on sedentary (i.e. sitting or lying), standing and active (i.e. walking, running, stair climbing or cycling).

Compositional means were used to describe the day-to-day pattern of work and leisure time physical behaviours [24, 33]. These were obtained by calculating the geometric mean of each physical behaviour of the respective compositions and then normalising the geometric means to the workers’ average accelerometer-derived daily work and leisure time (i.e. 450 min and 450 min, respectively). On non-workdays, the leisure time composition consisted of daily waking time, normalised to the workers’ average accelerometer-derived daily time spent awake (i.e. 960 min).

#### Calculation of pivot isometric log-ratios (ilrs) and model development

Daily work and leisure time-use compositions were expressed using pivot isometric log-ratio (ilr) coordinates [34]. The first pivot-coordinate was calculated as the normalised log-ratio of the first compositional part (i.e. behaviour), relative to the geometric mean of the remaining parts within the work and leisure time composition, respectively. The work and leisure time behaviours were sequentially rearranged to place each behaviour in the first position, where after the corresponding ilr-coordinate sets were computed. This way, the relative importance of each behaviour was sequentially represented in the first ilr-coordinate (ilr_1_) and used in the regression analysis. A detailed description of how the pivot-coordinates were calculated and model development is provided in Additional file 1.

The analysis was performed using two multivariate multilevel models. In both models, the outcome variables were the ilr-coordinates expressing the leisure time-use composition.

In Model 1, we investigated if leisure time physical behaviours differed between each day of the week (e.g. Monday, Tuesday, Wednesday, etc.) and between workday and non-workdays. Thus, day of the week and an interaction between day of the week and type of day (reference = non-workday) were entered as predictors in Model 1. Model 1 was fitted three times. This was done to isolate the association with one of the leisure time behaviours in relation to the others in the first ilr-coordinate (denoted by ilr_1_).

In Model 2, only workdays were considered as we investigated if work behaviours influenced day-to-day leisure time behaviours. The following predictors were entered in Model 2: the work time-use composition (i.e. work time sent standing, active and sedentary, expressed as ilr-coordinates) and an interaction term between day of the week and the work time-use composition. Model 2 was fitted six times to investigate the association between each part of the leisure time and work compositions, respectively. Of note, only results of the associations between the relative work time spent active and standing (as a proxies of physical work demands) and leisure time physical behaviours are shown. Results on the association between relative work time spent sedentary and day-to-day leisure time physical behaviours are shown in Additional file 2.

Both Model 1 and 2 were adjusted for the following covariates (reference in parenthesis for categorical variables): sex (men), smoking-status (smoker), BMI, and age. Model 2 was further adjusted for work duration. These covariates were chosen as potential confounders based on theoretical assumptions concerning their possible influence on day-to-day pattern of leisure time and work behaviours and work duration [13, 17]. In all models, Monday was selected as the reference category when entering the “type of the day” variable, as this is considered as the first day of the week in Denmark, where the International Standard ISO 8601 is followed.

#### Compositional isotemporal substitution analysis

Compositional isotemporal substitution analysis was used to provide meaningful interpretation of the expected change (in min/day) in leisure time-use compositions when time was reallocated between behaviours during work on workdays. This was done using the multivariate regression Model 2, stratified on each workday of the week. First, a “reference” leisure time-use composition (average daily leisure time spent sedentary, standing and active) was estimated for the workers’ mean work time-use composition (average min of work time spent sedentary, standing and active for that particular day). Second, new work time-use compositions were calculated where time (15, 30 and 45 min) had been reallocated between behaviours. This enabled us to express effect sizes as expected changes in leisure time behaviours in min/day. Note that results are only shown for workdays where the work behaviours were significantly associated with the leisure time behaviours. A detailed description of this method based on ilr linear regression with non-compositional and compositional outcomes can be found in Dumuid et al. [35] and Lund Rasmussen et al. [36].

All analyses were performed in R version 1.1.3 [37], using the *compositions* [38] and *MCMCglmm* [39] packages. We used the MCMCglmm package to conduct the multivariate multilevel analysis, following the guide provided by Baldwin et al. [40], by which a Bayesian approach with uninformative priors were used. The assumptions of normality and homoscedasticity of the residuals were assessed for all models by visual inspection of residuals versus predicted values and quantile-quantile plots.

#### Sensitivity analysis

A sensitivity analysis was conducted in which only workers with at least 2 days of measurements were included (*N* = 831). Results are shown in Additional file 3.

#### Assessment of potential selection bias

To identify potential selection bias, we compared the characteristics of the blue-collar workers included and excluded from the study. Differences between groups were investigated by calculating means and standard deviations or frequencies and percentages. Group differences were tested using t-test and Chi-squared statistics and a 5% significance threshold. Results are shown in Additional file 4.

## Results

### Study population characteristics

Table 1 shows the basic characteristics of the study population. Mean age was 44.9 (SD = 10.0) years, mean BMI was 27.2 kg/m2 (SD = 4.9), 45% were women and the majority were working within manufacturing (59%). On average, the participants had 4.1 (SD = 1.3) days with valid accelerometer measurements. Across all weekdays, the mean accelerometer-derived valid leisure and work hours were 7.4 (SD = 2.6) and 7.6 (SD = 2.1).

**Table 1:** Characteristics of the study population (*n* = 963)

Variable	N (%)	Mean (SD)
Age (years)	963 (100)	44.9 (10.0)
Seniority (years)	911 (95)	13.2 (10.4)
BMI in kg/m^2^	947 (98)	27.2 (4.9)
Aerobic capacity (ml O_2_/min/kg)	718 (75)	32.0 (9.0)
Alcohol consumption (units/week)	952 (99)	3.4 (5.1)
Days with accelerometer measurements	963 (100)	4.1 (1.3)
Accelerometer-derived leisure hours^a^	963 (100)	7.4 (2.6)
Accelerometer-derived work hours^a^	963 (100)	7.6 (2.1)
Sex		
Men	528 (55)	
Women	435 (45)	
Smoking-status		
Smoker	319 (33)	
Non-smoker	644 (67)	
Shift work		
Fixed day job	723 (75)	
Non-fixed day job	214 (22)	
Working sector		
Cleaning	175 (18)	
Manufacturing	569 (59)	
Transportation	69 (7)	
Health Service	19 (2)	
Assemblers	33 (3)	
Construction	40 (4)	
Garbage Collectors	29 (3)	
Mobile Plant Operators	11 (1)	
Other^b^	20 (2)	

^a^Across all weekdays.^b^Includes general office clerks and other elementary workers

### Compositional descriptive of day-to-day patterns in work and leisure time physical behaviours

Table 2 shows the compositional means of the day-to-day work and leisure time-use compositions on workdays and non-workdays. On workdays, the workers were predominantly sedentary during leisure time throughout the week. In contrast, most work time was spent standing on all working weekdays. On non-workdays, most time was spent sedentary throughout the week.

**Table 2:** Compositional means of leisure time and work physical behaviours on workdays and non-workdays

	Monday	Tuesday	Wednesday	Thursday	Friday	Saturday	Sunday
**Workdays**							
Work time behaviours (min/day (%))							
Sedentary	124 (28)	130 (29)	141 (31)	148 (33)	141 (31)	119 (26)	118 (26)
Standing	241 (53)	236 (52)	223 (50)	218 (48)	218 (48)	226 (50)	222 (50)
Active	85 (19)	84 (19)	86 (19)	84 (19)	91 (21)	105 (24)	110 (24)
Observations (n)^a^	154	332	457	544	388	77	47
Leisure time behaviours (min/day (%))^b^							
Sedentary	297 (66)	302 (67)	300 (67)	299 (66)	312 (69)	331 (74)	332 (74)
Standing	111 (25)	107 (24)	108 (24)	110 (25)	101 (23)	86 (19)	86 (19)
Active	42 (9)	41 (9)	42 (9)	41 (9)	37 (8)	33 (7)	32 (7)
Observations (n)^c^	184	362	498	569	423	103	58
**Non-workdays**							
Physical behaviours (min/day (%))							
Sedentary	648 (67)	655 (68)	630 (66)	625 (65)	612 (64)	609 (64)	602 (63)
Standing	228 (24)	227 (24)	244 (25)	242 (25)	256 (27)	263 (27)	267 (28)
Active	84 (9)	78 (8)	86 (9)	93 (10)	92 (9)	88 (9)	91 (9)
Observations (n)^a^	47	43	76	102	100	380	343

Active = walking, running, stair climbing and cycling. ^a^Workers with valid leisure time accelerometer measurements. ^b^Work time behaviours information only on workdays. ^c^Workers with valid work accelerometer measurements. Closure constant for leisure time composition was 450 min on workdays and 960 min on non-workdays based on the average accelerometer-derived leisure and non-work time. Closure constant for work time composition was 450 min based on the average accelerometer-derived work time

### Results of multilevel models

#### Leisure time physical behaviours on workdays vs. non-workdays (model 1)

Table 3 shows the results of model 1, investigating if the workers’ day-to-day leisure time physical behaviours differed between workdays and non-workdays. On Friday, Saturday and Sunday, the workers spent significantly more leisure time being sedentary on workdays compared with non-workdays (Table 3, ilr_1_(Sedentary_leisure) β_interaction_ = 0.26, 95% CI = (0.07; 0.44), ilr_1_(Sedentary_leisure) β_interaction_ = 0.34, 95% CI = (0.13; 0.55), and ilr_1_(Sedentary_leisure) β_interaction_ = 0.27, 95% CI = (0.04; 0.46), respectively). Moreover, the workers spent significantly less leisure time standing on Friday and Saturday on workdays compared with non-workdays (Table 3, ilr_1_(Standing_leisure) β_interaction_ = − 0.16, 95% CI = (− 0.32; − 0.02), and ilr_1_(Standing_leisure) β_interaction_ = − 0.21, 95% CI = (− 0.34; − 0.07), respectively).

**Table 3:** Association between day-to-day leisure time physical behaviours and weekday and workday

	Outcome: Leisure composition pivot coordinates		
Predictors	ilr_**1**_(Sedentary_leisure)	ilr_**1**_(Standing_leisure)	ilr_**1**_(Active_leisure)
	β (95% CI)	β (95% CI)	β (95% CI)
Weekday (Monday)			
Tuesday	−0.03 (− 0.25; 0.15)	0.04 (− 0.01; 0.18)	−0.02 (− 0.18; 0.13)
Wednesday	−0.03 (− 0.21; 0.16)	0.07 (− 0.05; 0.19)	−0.03 (− 0.16; 0.09)
Thursday	−0.09 (− 0.26; 0.10)	0.03 (− 0.08; 0.17)	0.05 (− 0.07; 0.17)
Friday	− 0.13 (− 0.27; 0.03)	0.11 (− 0.03; 0.24)	0.02 (− 0.11; 0.14)
Saturday	− 0.12 (− 0.26; 0.04)	**0.11 (0.01; 0.22)**	0.01 (− 0.11; 0.12)
Sunday	− 0.13 (− 0.28; 0.03)	**0.11 (0.01; 0.22)**	0.02 (− 0.01; 0.12)
Workday (yes)	− 0.07 (− 0.22; 0.01)	0.02 (− 0.11; 0.13)	0.05 (− 0.08; 0.17)
Workday (yes)*weekday (Monday)			
Tuesday	0.07 (− 0.11; 0.32)	− 0.07 (− 0.22; 0.09)	0.02 (− 0.12; 0.11)
Wednesday	0.05 (− 0.15; 0.26)	− 0.08 (− 0.21; 0.07)	0.02 (− 0.14; 0.17)
Thursday	0.12 (− 0.08; 0.28)	− 0.04 (− 0.18; 0.01)	−0.08 (− 0.24; 0.05)
Friday	**0.26 (0.07; 0.44)**	**−0.16 (− 0.32; − 0.02)**	−0.10 (− 0.25; 0.06)
Saturday	**0.34 (0.13; 0.55)**	**−0.21 (− 0.34; − 0.07)**	−0.14 (− 0.29; 0.01)
Sunday	**0.27 (0.04; 0.46)**	−0.13 (− 0.29; 0.02)	−0.14 (− 0.28; 0.01)

Active = walking, running, stair climbing, and cycling. ilr_1_ = first pivot coordinate, representing the relative importance of a leisure time physical behaviour (indicated in parenthesis) in relation to the others. Results based on multivariate multilevel models adjusted for sex, age, smoking-status and BMI. Total number of observations included = 3198. Bold indicates significant at *p* < 0.05. *indicates interaction term

These results suggested that the workers day-to-day leisure time behaviours differed between workdays and non-workdays and thus, further supported the investigation of whether day-to-day work physical behaviours caused this difference.

#### Standing work time and leisure time behaviours (model 2)

On all working weekdays, we observed a trend of relative standing work time to be positively associated with relative sedentary leisure time and negatively associated with relative standing and active leisure time. However, the associations were only statistically significant on Tuesday (Table 4; ilr_1_(Standing_leisure) β_interaction_ = − 0.17, 95% CI = (− 0.33; − 0.02)) and Sunday (ilr_1_(Sedentary_leisure) β_interaction_ = 0.93, 95% CI = (0.23; 1.60), ilr_1_(Standing_leisure) β_interaction_ = − 0.50, 95% CI = (− 0.93; − 0.12) and ilr_1_(Active_leisure) β_interaction_ = − 0.45, 95% CI = (− 0.91; − 0.01)). Specifically, the positive ilr_1_ beta-coefficient of 0.93 for Sedentary_leisure indicated that more standing work was associated with more sedentary leisure time on Sunday. In contrast, the negative ilr_1_ beta-coefficient of − 0.50 and − 0.45 for Standing_leisure and Active_leisure, respectively, showed that, on Sunday, more standing work was associated with less standing and active leisure time.

**Table 4:** Association between day-to-day leisure time physical behaviours, weekday, and relative standing work time

	Outcome: Leisure composition pivot coordinates		
Predictors	ilr_**1**_(Sedentary_leisure)	ilr_**1**_(Standing_leisure)	ilr_**1**_(Active_leisure)
	β (95% CI)	β (95% CI)	β (95% CI)
Weekday (Monday)			
Tuesday	−0.11 (− 0.30; 0.11)	0.12 (− 0.01; 0.30)	0.017 (− 0.13; 0.19)
Wednesday	− 0.04 (− 0.24; 0.17)	0.08 (− 0.07; 0.21)	0.002 (− 0.15; 0.15)
Thursday	− 0.05 (− 0.25; 0.17)	0.11 (− 0.03; 0.25)	− 0.04 (− 0.20;0.08)
Friday	−0.02 (− 0.20; 0.19)	0.09 (− 0.05; 0.23)	−0.03 (− 0.20; 0.09)
Saturday	0.06 (− 0.26; 0.32)	− 0.04 (− 0.23; 0.17)	−0.01 (− 0.19; 0.25)
Sunday	− 0.39 (− 0.87; 0.05)	0.24 (− 0.003; 0.51)	0.17 (− 0.10; 0.48)
ilr_1_(Standing_Work)	−0.08 (− 0.26; 0.17)	**0.18 (0.06; 0.32)**	−0.07 (− 0.24; 0.07)
ilr_1_(Standing_Work)*Weekday (Monday)			
Tuesday	0.13 (−0.13; 0.33)	**−0.17 (− 0.33; − 0.02)**	−0.01 (− 0.16; 0.18)
Wednesday	0.05 (− 0.18; 0.28)	−0.02 (− 0.25; 0.05)	0.002 (− 0.14; 0.17)
Thursday	0.06 (− 0.17; 0.27)	−0.11 (− 0.26; 0.02)	0.02 (− 0.14; 0.17)
Friday	0.21 (− 0.06; 0.39)	−0.15 (− 0.29; 0.02)	−0.02 (− 0.24; 0.06)
Saturday	0.21 (− 0.16; 0.59)	−0.09 (− 0.35; 0.16)	−0.15 (− 0.44; 0.11)
Sunday	**0.93 (0.23; 1.60)**	**−0.50 (− 0.93; − 0.12)**	**−0.45 (− 0.91; − 0.01)**

Active = walking, running, stair climbing, and cycling. ilr_1_ = first pivot coordinate, representing the relative importance of a work or leisure time physical behaviour (indicated in parenthesis) with respect to the others. Results based on multivariate multilevel models adjusted for sex, age, smoking-status and BMI. Total number of observations included = 1999. Bold indicates significant at *p* < 0.05, *indicates interaction term

#### Active work time and leisure time behaviours (model 2)

For relative active work time, we observed a negative association with relative sedentary leisure time and positive associations with relative standing and active leisure time throughout the week. These associations were only statistically significant on Tuesday (Table 5; ilr_1_(Standing_leisure) β_interaction_ = 0.20, 95% CI = (0.05; 0.42)), Friday (ilr_1_(Standing_leisure) β_interaction_ = 0.16, 95% CI = (0.01; 0.34)), and Sunday (ilr_1_(Sedentary_leisure) β_interaction_ = − 1.02, 95% CI = (− 1.67; − 0.33), ilr_1_(Standing_leisure) β_interaction_ = 0.56, 95% CI = (0.14; 1.04), and ilr_1_(Active_leisure) β_interaction_ = 0.47, 95% CI = (0.01; 0.97)).

**Table 5:** Association between day-to-day leisure time physical behaviours, weekday and relative active work time

	Outcome: Leisure composition pivot coordinates		
Predictors	ilr_**1**_(Sedentary_leisure)	ilr_**1**_(Standing_leisure)	ilr_**1**_(Active_leisure)
	β (95% CI)	β (95% CI)	β (95% CI)
Weekday (Monday)			
Tuesday	−0.13 (− 0.29; 0.14)	0.13 (− 0.01; 0.28)	0.003 (− 0.16; 0.16)
Wednesday	− 0.06 (− 0.24; 0.12)	0.08 (− 0.07; 0.21)	− 0.02 (− 0.154; 0.14)
Thursday	−0.06 (− 0.25; 0.11)	0.12 (− 0.04; 0.24)	−0.05 (− 0.18; 0.10)
Friday	−0.05 (− 0.27; 0.11)	0.09 (− 0.03; 0.26)	−0.05 (− 0.19; 0.10)
Saturday	0.04 (− 0.27; 0.31)	−0.03 (− 0.23; 0.19)	−0.01 (− 0.12; 0.22)
Sunday	**−0.41 (− 0.82; − 0.05)**	**0.26 (0.01; 0.55)**	0.14 (− 0.13; 0.44)
ilr_1_(Active_Work)	0.12 (− 0.09; 0.31)	**−0.21 (− 0.38; − 0.04)**	0.09 (− 0.05; 0.26)
ilr_1_(Active_Work)*Weekday (Monday)			
Tuesday	−0.21 (− 0.48; 0.04)	**0.20 (0.05; 0.42)**	0.02 (− 0.16; 0.17)
Wednesday	−0.08 (− 0.27; 0.12)	0.11 (− 0.02; 0.29)	−0.03 (− 0.21; 0.13)
Thursday	−0.02 (− 0.32; 0.16)	0.15 (− 0.04; 0.30)	−0.04 (− 0.21; 0.10)
Friday	−0.19 (− 0.45; 0.01)	**0.16 (0.01; 0.34)**	0.04 (− 0.10; 0.22)
Saturday	−0.28 (− 0.72; 0.12)	0.03 (− 0.30; 0.28)	0.25 (− 0.02; 0.51)
Sunday	**− 1.02 (− 1.67; − 0.33)**	**0.56 (0.14; 1.04)**	**0.47 (0.01; 0.97)**

Active = walking, running, stair climbing, and cycling. ilr_1_ = first pivot coordinate, representing the relative importance of a work or leisure time physical behaviour (indicated in parenthesis) with respect to the others. Results based on multivariate multilevel models adjusted for sex, age, smoking-status and BMI. Total number of observations included = 1999. Bold indicates significant at *p* < 0.05, *indicates interaction term

### Results of compositional isotemporal substitutions

Tables 6 and 7 show the results of the compositional isotemporal substitution. Note that only results for reallocations on Tuesday and Sunday are shown, as we observed the strongest relationship between work and leisure time behaviours on these working weekdays.

**Table 6:** Expected difference in leisure time behaviours following reallocation between work physical behaviours on Tuesday

	Estimated Leisure Time Behaviours					
Work Time Behaviours	Sedentary		Standing		Active	
	Min/day	Δ	Min/day	Δ	Min/day	Δ
*Increasing sedentary work time*						
Average work time-use composition	309		100		42	
+ 15 min sedentary	309	0	100	0	42	0
+ 30 min sedentary	310	+ 1	99	−1	42	0
+ 45 min sedentary	310	+ 1	99	−1	42	0
*Increasing standing work time*						
Average work time-use composition	309		100		42	
+ 15 min standing	309	0	100*	0	42	0
+ 30 min standing	310	+ 1	99*	−1	42	0
+ 45 min standing	309	+ 2	99*	−1	40	−2
*Increasing active work time*						
Average work time-use composition	309		100		42	
+ 15 min active	308*	−1	100*	0	43	+ 1
+ 30 min active	306*	−3	101*	+ 1	44	+ 2
+ 45 min active	305*	+ 4	102*	+ 2	44	+ 2

Active = walking, running, stair climbing, and cycling. Results based on multivariate models adjusted for sex, age, smoking-status, BMI and work duration (results shown in Tables 4 and 5). * indicates significant at *p* < 0.05

**Table 7:** Expected difference in leisure time behaviours following reallocation between work physical behaviours on Sunday

	Estimated Leisure Time Behaviours					
Work Time Behaviours	Sedentary		Standing		Active	
	Min/day	Δ	Min/day	Δ	Min/day	Δ
*Increasing sedentary work time*						
Average work time-use composition	326		91		33	
+ 15 min sedentary	327	+ 1	90	−1	33	0
+ 30 min sedentary	327	+ 1	90	−1	33	0
+ 45 min sedentary	328	+ 2	90	−1	32	− 1
*Increasing standing work time*						
Average work time-use composition	326		91		33	
+ 15 min standing	335*	+ 9	85*	−6	30*	−3
+ 30 min standing	344*	+ 18	80*	−11	26*	−7
+ 45 min standing	352*	+ 26	75*	−16	24*	−9
*Increasing active work time*						
Average work time-use composition	326		91		33	
+ 15 min active	314*	−12	98*	+ 7	38*	+ 5
+ 30 min active	301*	−25	106*	+ 15	43*	+ 10
+ 45 min active	289*	−37	113*	+ 22	48*	+ 15

Active = walking, running, stair climbing, and cycling. Results based on multivariate models adjusted for sex, age, smoking-status, BMI and work duration (results shown in Tables 4 and 5). * indicates significant at *p* < 0.05

On Tuesday (Table 6), reallocating 30 min work time to standing from the remaining work behaviours was associated with 1 min less standing leisure time. Reallocating 30 min to active work time was associated with 3 min less sedentary leisure time and 1 min more standing leisure time.

On Sunday (Table 7), reallocating 30 min work time to standing from the remaining work behaviours was associated with 18 min more sedentary time, 11 min less standing time, and 7 min less active time during leisure. Reallocating 30 min to active work time from the remaining work behaviours was associated with 25 min less sedentary time, a 15 min increase in standing time and a 10 min increase active time during leisure.

### Sensitivity analysis

Results of sensitivity analysis among workers with at least 2 days of valid accelerometer data corresponded to those from the primary analyses (results shown in Additional file 3).

### Assessment of potential selection bias

Comparison of the characteristics of blue-collar workers excluded and included in the current study is shown in Additional file 4. Blue-collar workers excluded from the study sample (*n* = 244) had, on average, a lower seniority (mean = 11.6 years, SD = 10.2) and aerobic capacity (mean = 30.0, SD = 7.0) and a higher proportion was women and cleaners (24%) or transporters (9%) compared to those included (*n* = 963).

## Discussion

In this study, we investigated the day-to-day pattern of leisure time physical behaviours among blue-collar workers on workdays and non-workdays. Furthermore, we assessed the association between day-to-day work physical behaviours and leisure time physical behaviours. The workers were primarily sedentary during leisure throughout the week on both workdays and non-workdays. Moreover, the workers were more sedentary at leisure during the weekend on workdays compared to non-workdays. Regarding the association between work and leisure time physical behaviours, standing work time was positively associated with sedentary leisure time and negatively associated with standing and active leisure time. The opposite direction was found for the association between active work time and leisure time physical behaviours.

The overall finding of our study is that over the course of a week, low SES adults were predominantly sedentary during leisure, while most work time was spent standing or active. To our knowledge, this is the first study to investigate the domain-specific pattern of day-to-day physical behaviours among low SES adults, while taking the inter-dependency between daily time-use into account using CoDA. Nevertheless, our results correspond to those reported in reviews finding leisure time PA to be less prevalent and work PA to be more prevalent among low SES adults compared with high SES adults when considering weekly averages [15, 41]. Moreover, accelerometer-based studies have consistently found low SES adults to be more active on workdays compared to non-workdays. One study found Swiss workers with manual jobs (e.g. craftsmen, machine operators and labourers) to be more active on workdays than non-workdays [42]. Another study reported adults with low educational level as less likely to be active during the weekend (i.e. non-workdays) and more likely to be active during the weekdays (i.e. workdays) compared with those with high educational level [13]. Finnish low-level occupational groups (e.g. cleaners, plumbers and construction workers) and Australian blue-collar workers have been found to be more sedentary and take less steps on weekends (i.e. non-workdays) compared with weekdays (i.e. workdays) [14, 43]. Taken together, these findings suggest that low SES adults derive the greatest proportion of day-to-day PA from work activities. Consequently, when only considering leisure time physical behaviours, low SES adults could erroneously be perceived to be “couch potatoes”. However, taking work physical behaviours into consideration, this population group might also be “work warriors”.

We further investigated whether the workers’ work physical behaviours was associated with their high amount of sedentary leisure time throughout the week. For example, on Sunday, reallocating 30 min to standing work time was associated with an 18 min increase in sedentary leisure time, an 11 min decrease in standing leisure time, and a 7 min decrease in active leisure time. This finding could be explained by the fact that standing for prolonged, uninterrupted periods increase blood pooling in the legs which can cause swelling, pain and muscle fatigue in the lower extremities [44, 45]. Consequently, workers with much standing work time could perceive to have an increased need to compensate with more sedentary time and less physical activities when coming home from work [46]. We have previously assessed the association between percentage work hours spent standing and percentage leisure time spent sedentary over consecutive workdays within the same population [47]. However, contrary to the present study, no relationship between occupational standing and sedentary leisure time was observed. One explanation for the discrepancy between the two studies could be that we did not consider the co-dependency between work and leisure time physical behaviours as done in the current study.

Counter to standing work time, active work was associated with less sedentary and more standing and active leisure time. For example, reallocating 30 min to active work time was associated with a 25 min decrease in sedentary leisure time, a 15 min increase in standing leisure time, and a 10 min increase in active leisure time on Sunday. This result is in line with one study finding a positive relationship between accelerometer-derived time spent on moderate-to-vigorous physical activity at work and leisure among 233 adults [48]. In contrast, another study based on accelerometer measurement from 112 adults found no difference in leisure physical activities between those with low and high work activity levels [49]. Thus, the relationship between work and leisure time physical behaviours among low SES adults remains unclear. We encourage future research on this topic based on technical measurements and considering the inter-dependency between time spent in daily behaviours.

### Practical implications

Most physical activity interventions focus on individual factors such as motivation and self-efficacy [50]. This could be a result of cognitive social theories dominating the behavioural research on physical activity for decades. Accordingly, this research field has been shaped by the assumption that being active or inactive are deliberate choices. However, motivating individuals to be active without considering barriers is likely to be ineffective, particularly for low SEP adults. In fact, interventions that solely rely on individual agency to increase physical activity levels risk widening social inequalities due to higher reach and effectiveness among high SEP adults [51]. Instead, interventions should both address modifiable barriers, such as work factors, that limit opportunities for being active, while protecting and enhancing factors that enable and encourage this behaviour [52].

Based on the findings of this study, standing at work could be a barrier for low SES adults to be physically active during leisure time. Thus, we encourage future intervention studies to investigate the effectiveness of reducing this physical work demand on leisure time behaviours. For workers for whom work time spent standing cannot be altered, it will be important to investigate the health effect and feasibility of different combinations of leisure time spent on sedentary (to promote post-work recovery) and health-enhancing activities over the course of a week.

Although the estimated changes in leisure time physical behaviours might appear small (e.g. a 7 min decrease in active leisure time when increasing standing work time), the found changes in leisure time should be considered in relation to the overall leisure time physical behaviours. As this group of adults was predominantly sedentary during leisure time, any additional trade-off between sedentary and active leisure time could have important long-term health implications. Moreover, it should be noted that this study was conducted in a high-income country. Considering that work has been found to be a greater contributor to moderate-to-vigorous physical activity levels in lower compared with higher income countries [53], we would expect our findings to be more prominent among lower income countries.

### Strengths and limitations

This study was based on technical measurements, which enabled accurate information on daily time spent in physical behaviours at work and during leisure time. Particularly, the use of the Acti4 program to measure physical behaviours was a strength of the study as it enabled distinction between specific physical behaviours, which fall within the same intensity, such as standing and walking, with high sensitivity and specificity [30, 31]. The use of multivariate multilevel models was another strength by allowing analyses which included the repeated measurements as well as multiple outcomes for each participant.

Given the close link between SES and work and leisure time physical behaviours [15, 41, 54], there is always a risk of socioeconomic confounding if SES is not appropriately accounted for. While there is no single best indicator of SES, occupational class is well-acknowledged to reflect social standing among working adults [55, 56]. Accordingly, the use of a study population consisting of adults who were homogenous in occupational class was a strength as it limited the possibility of socioeconomic confounding. Nevertheless, the lack of information on other measures of socioeconomic status such as education and income was a limitation of our study.

This study was based on blue-collar workers recruited through low profit workplaces from a range of different sectors and locations in Denmark to ensure a study population representative of typical blue-collar workers [57, 58]. Accordingly, the characteristics of the study population in the DPhacto study has been considered as representative for the target population [57]. Nevertheless, we observed that the blue-collar workers excluded in the study had a lower aerobic capacity compared to those included. A high aerobic capacity can enhance the workers’ ability to sustain their physical work, and thereby decrease levels of post-work fatigued [59]. Accordingly, the found differences could have underestimated the relationship between work and leisure time physical behaviours.

The cross-sectional design of this study hinders causal inference. The workers included in the NOMAD and DPhacto studies were asked to wear the accelerometers for at least two workdays. This was to enhance the feasibility of the workers to participate in the study but might have compromised the representativeness of the measurements. Most of the workers had the accelerometers mounted on Monday or Tuesday. Thus, it is possible that the workers behaved differently these days because they wore accelerometers. Moreover, participants with heavy physical work or a very active leisure time could have experienced issues with wearing the accelerometer, causing them to take off the devices at an earlier stage than less active participants. However, the sensitivity analysis excluding those workers with only 1 day of valid accelerometer data revealed results similar to that of the primary analysis.

We lacked information about the context in which day-to-day physical behaviours were performed. Thus, we are unable to state if active leisure time spent was spent on active transport; health-promoting planned activities such as sports; or on domestic work. The adults included in this study spent less than 1% of daily work and leisure time spent in activities which could be considered of higher intensities (i.e. running, stair climbing, and biking). Thus, we decided to combine these activities with walking, which could have attenuated some of the tested associations. Finally, we did not include time in bed at night in the analysis, which we encourage future studies on day-to-day patterns of daily physical behaviours to consider.

## Conclusions

We found low SES adults to be primarily sedentary during leisure time, while work time was spent mostly standing or active. Thus, this group of adults could be characterised as ‘work warriors’. This highlights the need of differentiating between work and leisure time when assessing daily behavioural patterns among low SES adults. Standing work time was associated with a more sedentary leisure time, whereas the opposite was found for the association between active work time and leisure time physical behaviours. Accordingly, we recommend strategies for increasing health-enhancing leisure time PA among low SES adults to consider the potential influence of work physical behaviours.

## Supplementary Information

**Additional file 1.** Calculation of pivot-coordinates and model development. Detailed description of how pivot-coordinates were calculated and models developed.

**Additional file 2.** Association between day-to-day leisure time physical behaviours, weekday and relative sedentary work time. Results on the association between day-to-day relative work time spent sedentary and leisure time physical behaviours.

**Additional file 3.** Sensitivity analyses. Results of sensitivity analyses in which only workers with at least two days of measurements were included.

**Additional file 4.** Assessment of potential selection bias. Comparison of baseline characteristics between blue-collar workers excluded from and included in the study.

## Data Availability

The datasets supporting the conclusions of this article are available at the Danish National Archives, https://www.sa.dk/en/k/about-us.

## Abbreviations

DPhacto: Danish PHysical ACTivity cohort with Objective measurements
NOMAD: New Method for Objective Measurements of Physical Activity in Daily Living
PA: physical activity
SES: socioeconomic status
WHO: World Health Organization
