# Supplementary material for: Day-to-day pattern of work and leisure time physical behaviours: are low socioeconomic status adults couch potatoes or work warriors?
Source: BMC Public Health. 2021 Jul 7;21:1342. doi: 10.1186/s12889-021-11409-0 (PMC8265073; doi:10.1186/s12889-021-11409-0)
Supplement: Supplementary file 1 — Additional file 1. Calculation of pivot-coordinates and model development. Detailed description of how pivot-coordinates were calculated and models developed. [file 12889_2021_11409_MOESM1_ESM.docx]

**ADDITIONAL FILE 1**

## Ilr coordinates for work and leisure composition

The day-to-day physical behaviours during leisure time and at work were defined as 3-part compositions and expressed isometric log-ratio (ilr) coordinates. Following [1,2], for a $D-$part composition $\mathbf{x}=(x_{1},\ldots,x_{D})$ we can obtain a real vector $\mathbf{z}=(z_{1},z_{2},\ldots, z_{D-1})$ of $D-1$ ilr-coordinates, where

| $z_{j=}\sqrt{\frac{D-j}{D-j+1}}\ln\frac{x_{j}}{\sqrt[D-j]{\prod_{k=j+1}^{D} x_{k}}}, j=1,\ldots, D-1$ | (A1) |
| --- | --- |

This is a particular choice of ilr-coordinates is known as pivot coordinates [3], by which all relative information about the first part of the composition ($x_{1}$) is included in the first ilr-coordinate ($z_{1}$). This way, $z_{1}$ represents the relative importance of the part $x_{1}$ with respect to an (geometric) average of the remaining parts in the composition. The remaining ilr-coordinates ($z_{2}, z_{3},\ldots, z_{D}$) contain no information about the first part of the composition.

For example, pivot coordinates of the leisure time composition (i.e. the outcome of this study) were computed for each time point (*t*) as

| $y_{1it}^{*}=\sqrt{\frac{2}{3}}\ln\left( \frac{{sedentary}_{{leis}_{it}}}{\sqrt[3]{{standing}_{{leis}_{it}}*{active}_{{leis}_{it}}}} \right)$  $y_{2it}^{*}=\sqrt{\frac{1}{2}}\ln\left( \frac{{standing}_{{leis}_{it}}}{\sqrt[2]{{active}_{{leis}_{it}}}} \right)$ | (A2) |
| --- | --- |

Giving rise to a leisure ilr-coordinate vector $ilr\left( \boldsymbol{Y}_{it} \right)=\left( \begin{matrix} y_{1it}^{*} \\ y_{2it}^{*} \end{matrix} \right)$ for up to seven time points (*t*=0,…,6) for each worker (*i*).

Similarly, pivot coordinates of the work composition (i.e. the exposures of interest in this study) were computed for each time point (*t*) as

| $z_{1it}^{*}=\sqrt{\frac{2}{3}}\ln\left( \frac{{standing}_{{work}_{it}}}{\sqrt[3]{{active}_{{work}_{it}}*{sedentary}_{{work}_{it}}}} \right)$  $z_{2it}^{*}=\sqrt{\frac{1}{2}}\ln\left( \frac{{sedentary}_{{work}_{it}}}{{active}_{{work}_{it}}} \right)$ | (A3) |
| --- | --- |

Giving rise to a vector for the work ilr-coordinate vector $ilr\left( \boldsymbol{Z}_{it} \right)=\left( \begin{matrix} z_{1it}^{*} \\ z_{2it}^{*} \end{matrix} \right)$ for up to seven time points (*t*=0,…,6) for each worker (*i*).

With the objective of investigating the association between the leisure time physical behaviours and the day of the week and type of day (i.e. nonwork vs workday), the parts were sequentially rearranged within the leisure time composition to place each part at the first position. Moreover, to assess the association between parts of the work and leisure composition, the parts within the work composition were also sequentially rearranged.

Given the co-dependency between the parts of the leisure time composition at each time point, this relationship should be taken into account when modelling the association between daily work and leisure time physical behaviours. This was possible using a multivariate multilevel model following [4,5].

## The univariate multilevel model

A generic unconditional univariate multilevel model has two levels:

| Level 1: | | $y_{it}=\beta_{0t}+e_{it}$, | (A4) |
| --- | --- | --- | --- |
| Level 2: | $\beta_{0t}=y_{00}+u_{0t}$. | | (A5) |

Substituting A5 into A4 produces the following combined univariate multilevel model:

| $y_{it}=y_{00}+u_{0t}+e_{it}$, | (A6) |
| --- | --- |

where $\beta_{0j}$ is the mean of the outcome at time point *t* and $y_{00}$ is the grand mean of the outcome across all individuals. $e_{ij}$ is the level-1 residual (within-person deviation) for individual *i* at time point *t* and $u_{0t}$ is the level-2 residuals (between-person deviation) assessing the deviation between the grand mean and the time point specific mean. Moreover, the following variance components are estimated

$e_{ij}\sim N\left( 0,\sigma_{e}^{2} \right)$ *and* $u_{0j}\sim N(0,\sigma_{0}^{2})$*,*

where $\sigma_{e}^{2}$ and $\sigma_{0}^{2}$ represent the residual and intercept variance, respectively.

## The conditional univariate multilevel model

We can further extend model A6 to include level-1 and level-2 predictors:

| Level 1: | $y_{it}=\beta_{0t}+\beta_{1t}x_{it}+e_{it}$, | (A7) | |  |
| --- | --- | --- | --- | --- |
| Level 2: | $\beta_{0t}=y_{00}+y_{01}z_{t}+u_{0t}$. | | (A8) | |

Substituting A8 into A7 produces the following combined conditional univariate multilevel model:

| $y_{it}=y_{00}+\beta_{1t}x_{it}+y_{01}z_{t}+u_{0j}+e_{it}$, | (A9) |
| --- | --- |

where $x_{it}$ represent a level-1 predictor, i.e. a factor for individual *i* at time point *t,* and $z_{t}$ represent a level-2 predictor, i.e. a factor at time point *t.* The remaining model parameters have the same interpretation as in model A6 and the same variance components are estimated.

## The unconditional multivariate multilevel model

The univariate multilevel model A6 can be extended to a multivariate case, suitable for the current study where each individual had multiple outcome variables (i.e. two pivot coordinates to express the 3-part leisure time composition) measured at several consecutive days.

Let $y_{kit}^{*}$ be a value for individual *i* at time point *t* for the *k-*th outcome variable and $y$ is considered as a single variable with its measurement defined by its subscripts. Next, dummy variables $\delta_{1},\ldots,\delta_{k}$ can be defined for each pivot coordinate.

Similar to the univariate multilevel model A6, the multivariate model has two levels:

| Level 1: | $y_{kit}^{*}=\sum_{k} \delta_{k}(\beta_{0tk}+e_{itk})$, | (A10) |
| --- | --- | --- |

| Level 2: | $\beta_{0tk}=y_{00k}+u_{0tk}$. | (A11) |
| --- | --- | --- |

By substitution A11 into A10, the combined unconditional multivariate multilevel model is defined as:

| $y_{kit}^{*}=\sum_{k} \delta_{k}\left( y_{00k}+u_{0tk}+e_{itk} \right)$  $=\sum_{k} \left( y_{00k}\delta_{k}+{\delta_{k}u}_{0tk}+\delta_{k}e_{itk} \right)$, | (A12) |
| --- | --- |

where $\beta_{0tk}$ is mean of the *k*-th outcome variable at time point *t* and $y_{00k}$ is the grand mean of the outcome across all individuals for the *k*-th outcome. $e_{itk}$ is the level-1 residual (within-person deviation) for individual *i* at time point *t* for *k*-th outcome. Finally, $u_{0tk}$ it the level-2 residuals (between-person deviation) assessing the deviation between the grand mean and the time point specific mean for *k*-th outcome.

The multivariate multilevel model enables estimations of residual variance for each outcome and of variances and covariance for random parameters between outcome variables. For example, when assessing the effect of workday on the leisure time composition (i.e. vector $ilr\left( \boldsymbol{Y} \right)$) a total of 2 outcomes were modelled (k=2) and the following random effects were estimated:

$\left[ \begin{matrix} e_{i_{k=1}} \\ e_{i_{k=2}} \end{matrix} \right]\sim MVN\left( 0,\Omega_{R} \right), \Omega_{R}=\left[ \begin{matrix} \sigma_{e_{k=1}}^{2} & \\ \sigma_{e_{k=1}e_{k=2}} & \sigma_{e_{k=2}}^{2} \end{matrix} \right]$,

where $e_{i_{k=1}}$ and $e_{i_{k=2}}$ are the (level-1) residual errors for each pivot coordinate and $\Omega_{R}$ is the covariance between these residuals.

Moreover, assuming multivariate normal distribution, the following random effects were estimated to express the covariance between any combinations of random intercepts. For example, when modelling the effect of workday on the leisure time composition, the following covariance parameters were estimated:

$$\left[ \begin{matrix} u_{0i_{k=1}} \\ u_{0i_{k=2}} \end{matrix} \right]\sim MVN\left( 0,\Omega_{G} \right), \Omega_{G}=\left[ \begin{matrix} \sigma_{0_{k=1}}^{2} & \\ \sigma_{0_{k=1k=2}}^{2} & \sigma_{0_{k=2}}^{2} \end{matrix} \right],$$

where $u_{0i_{k=1}}$and $u_{0i_{k=2}}$ are the (level-2) residual errors for each pivot coordinate and $\Omega_{R}$ is the covariance for the pair of outcomes. Thus, $\sigma_{0_{k=1k=2}}^{2}$ expresses the covariance between intercepts of pivot coordinate $y_{1it}^{*}$ and $y_{2it}^{*}$.

## The conditional multivariate multilevel model

The conditional univariate model A9 can be extended to a multivariate case, including level 1 and level 2 predictors:

| Level 1: | | $y_{kit}^{*}=\sum_{k} \delta_{k}(\beta_{0tk}{+\beta}_{1t}x_{it}+e_{itk})$ | (A13) |
| --- | --- | --- | --- |
| Level 2: | $\beta_{0tk}=y_{00k}+y_{01k}z_{tk}+u_{0tk}$. | | (A14) |

Substituting A7 into A6 produces the following combined conditional multivariate multilevel model:

| $y_{kit}^{*}=\sum_{k} \delta_{k}\left( y_{00k}+\beta_{1tk}x_{itk}+y_{01k}z_{tk}+u_{0tk}+e_{itk} \right)$  $=\sum_{k} \left( y_{00k}\delta_{k}+\beta_{1tk}\delta_{k}x_{itk}+y_{01k}\delta_{k}z_{tk}+{\delta_{k}u}_{0tk}+{\delta_{k}e}_{itk} \right)$, | (A15) |
| --- | --- |

where $\beta_{1tk}\delta_{k}x_{itk}$ represent a level-1 predictor for individual *i* at time point *t* for the k-th outcome. $y_{01k}\delta_{k}z_{tk}$ represent a level-2 predictor at time point *t* or the k-th outcome. The same variance components are estimated as with model A12.

## Application to the current study

In the current study we assessed the association between of 1) day of the week, 2) type of day (i.e. workday vs. non-workday), and 3) the work time-use composition (expressed as pivot-coordinates, e.g. vector$ilr\left( \boldsymbol{Z}_{it} \right)$ above) and the leisure time-use composition (expressed as pivot-coordinates, e.g. vector$ilr\left( \boldsymbol{Y}_{it} \right)$ above). The analysis was performed in multiple steps using conditional multivariate multilevel models (A15).

In model 1, day of the week and an interaction between day of the week (i.e. weekday) and type of day (i.e. daytype, reference=non-workday) were entered as a level-2 predictors. Moreover, the following level-2 predictors were included as potential confounders: (reference in parenthesis for categorical variables): sex (men), smoking-status (smoker), BMI, and age. Thus, model 1 was defined as shown in A16. Model 1 was fitted 3 times, sequentially rearrange the first part of the leisure time composition as described above.

| $y_{kit}^{*}=\sum_{k} \delta_{k}\left( y_{00k}+y_{01k}{weekday}_{tk}+y_{02k}{daytype}_{tk}+y_{03k}{sex}_{tk} +y_{04k}{smoking}_{tk}+y_{05k}{BMI}_{tk}{+y_{06k}{age}_{tk}+u}_{0tk}+e_{itk} \right)$ | (A16) |
| --- | --- |

In model 2, the following level-2 predictors were entered: work duration, the work time-use composition and interaction terms between day of the week and the work time-use composition, respectively. Work duration was calculated as the log of total accelerometer-derived work time [6]. Model 2 was adjusted for the same level-2 predictors as model 1. Thus, model 2 was defined as shown in A17.

| $y_{kit}^{*}=\sum_{k} \delta_{k}\left( y_{00k}+y_{01k}{weekday}_{tk}+y_{02k}{workduration}_{tk}+y_{04k}z_{1tk}^{*}+y_{05k}z_{2tk}^{*}+y_{06k}z_{1tk}^{*}{weekday}_{tk}+y_{07k}z_{2tk}^{*}{weekday}_{tk}+y_{08k}{sex}_{tk} +y_{09k}{smoking}_{tk}+y_{10k}{BMI}_{tk}{+y_{11k}{age}_{tk}+u}_{0tk}+e_{itk} \right)$ | (A17) |
| --- | --- |

Where $z_{1tk}^{*}$ and $z_{2tk}^{*}$represent the first and second pivot-coordinate of the work composition at time point *t* for the k-th outcome. Model 2 was fitted six times, each time sequentially rearrange the first part of either the work or leisure time composition. This enabled assessment of the association between each part of the two compositions.

**References**1. Hron K, Filzmoser P, Thompson K. Linear regression with compositional explanatory variables. Journal of Applied Statistics. 2012;39:1115–28.

2. Chastin SFM, Palarea-Albaladejo J, Dontje ML, Skelton DA. Combined Effects of Time Spent in Physical Activity, Sedentary Behaviors and Sleep on Obesity and Cardio-Metabolic Health Markers: A Novel Compositional Data Analysis Approach. PLOS ONE. 2015;10:e0139984.

3. Hron K, Filzmoser P, Caritat P de, Fišerová E, Gardlo A. Weighted Pivot Coordinates for Compositional Data and Their Application to Geochemical Mapping. Math Geosci. 2017;49:797–814.

4. MacCallum RC, Kim C, Malarkey WB, Kiecolt-Glaser JK. Studying Multivariate Change Using Multilevel Models and Latent Curve Models. Multivariate Behav Res. 1997;32:215–53.

5. Goldstein H. Multilevel Statistical Models. Chichester, UK: John Wiley & Sons, Ltd; 2010.

6. Vera Pawlowsky-Glahn, Juan José Egozcue, David Lovell. Tools for compositional data with a total. Statistical Modelling. 2015;15:175–90.
