# Supplementary material for: Day-to-day pattern of work and leisure time physical behaviours: are low socioeconomic status adults couch potatoes or work warriors?
Source: BMC Public Health. 2021 Jul 7;21:1342. doi: 10.1186/s12889-021-11409-0 (PMC8265073; doi:10.1186/s12889-021-11409-0)
Supplement: Supplementary file 2 — Additional file 2. Association between day-to-day leisure time physical behaviours, weekday and relative sedentary work time. Results on the association between day-to-day relative work time spent sedentary and leisure time physical behaviours. [file 12889_2021_11409_MOESM2_ESM.docx]

**ADDITIONAL FILE 2**

**Table A1.** Association between day-to-day leisure time physical behaviours, weekday and relative sedentary work time.

|  | **Outcome: Leisure composition pivot coordinates** | | |
| --- | --- | --- | --- |
| **Predictors** | **ilr_1_(Sedentary_Leisure)** | **ilr_1_(Standing_Leisure)** | **ilr_1_(Active_Leisure)** |
|  | β (95 % CI) | β (95 % CI) | β (95 % CI) |
| Weekday (Monday) |  |  |  |
| Tuesday | -0.14 (-0.33 ; 0.05) | 0.11 (-0.02 ; 0.28) | 0.01 (-0.15 ; 0.17) |
| Wednesday | -0.07 (-0.22 ; 0.12) | 0.07 (-0.06 ; 0.22) | -0.01 (-0.17 ; 0.14) |
| Thursday | -0.07 (-0.26 ; 0.10) | 0.11 (-0.06 ; 0.22) | -0.04 (-0.22 ; 0.10) |
| Friday | -0.05 (-0.22 ; 0.12) | 0.08 (-0.07 ; 0.21) | -0.04 (-0.20 ; 0.12) |
| Saturday | 0.04 (-0.22 ; 0.32) | -0.04 (-0.26 ; 0.21) | -0.01 (-0.23 ; 0.22) |
| Sunday | **-0.39 (-0.75 ; -0.02)** | 0.24 (-0.08 ; 0.58) | 0.14 (-0.13 ; 0.46) |
| ilr_1_(Sedentary_Work) | -0.02 (-0.10 ; 0.07) | 0.03 (-0.04 ; 0.10) | -0.01 (-0.07 ; 0.08) |
| ilr_1_(Sedentary_Work)*Weekday (Monday) | | | |
| Tuesday | 0.06 (-0.03 ; 0.17) | -0.03 (-0.11 ; 0.04) | -0.03 (-0.11 ; 0.05) |
| Wednesday | 0.01 (-0.11 ; 0.09) | -0.01 (-0.09 ; 0.06) | 0.004 (-0.07 ; 0.08) |
| Thursday | 0.03 (-0.09 ; 0.12) | -0.04 (-0.12 ; 0.04) | 0.01 (-0.06 ; 0.09) |
| Friday | -0.03 (-0.14 ; 0.06) | -0.01 (-0.08 ; 0.08) | 0.04 (-0.03 ; 0.13) |
| Saturday | 0.04 (-0.14 ; 0.22) | 0.07 (-0.07 ; 0.23) | -0.12 (-0.26 ; 0.03) |
| Sunday | 0.06 (-0.17 ; 0.27) | -0.03 (-0.19 ; 0.14) | -0.05 (-0.21 ; 0.13) |

*Active=walking, running, stair climbing, and cycling. ilr_1_ = first pivot coordinate, representing the relative importance of a work or leisure time physical behaviour (indicated in parenthesis) with respect to the others. Results based on multivariate multilevel models adjusted for sex, age, smoking-status and BMI. Total number of observations included = 1999. Bold indicates significant at p <0.05, *indicates interaction term.*
