# Supplementary material for: Day-to-day pattern of work and leisure time physical behaviours: are low socioeconomic status adults couch potatoes or work warriors?
Source: BMC Public Health. 2021 Jul 7;21:1342. doi: 10.1186/s12889-021-11409-0 (PMC8265073; doi:10.1186/s12889-021-11409-0)
Supplement: Supplementary file 3 — Additional file 3. Sensitivity analyses. Results of sensitivity analyses in which only workers with at least two days of measurements were included. [file 12889_2021_11409_MOESM3_ESM.docx]

**Additional file 3**

Tables below show the results of sensitivity analyses in which only workers with at least two days of measurements were included (N=831).

**Table A1.** Association between day-to-day leisure time physical behaviours, weekday and relative standing work time.

| **Outcome: Leisure composition pivot coordinates** | | | | |
| --- | --- | --- | --- | --- |
| **Predictors** | **ilr_1_(Sedentary_Leisure)** | **ilr_1_(Standing_Leisure)** | **ilr_1_(Active_Leisure)** | |
|  | β (95 % CI) | β (95 % CI) | β (95 % CI) | |
| Weekday (Monday) |  |  |  | |
| Tuesday | -0,12 (-0,37 ; 0,05) | 0,15 (0,004 ; 0,28) | -0,003 (-0,16 ; 0,16) | |
| Wednesday | -0,05 (-0,24 ; 0,14) | 0,09 (-0,03 ; 0,23) | -0,02 (-0,15 ; 0,14) | |
| Thursday | -0,04 (-0,23 ; 0,16) | 0,11 (-0,03 ; 0,26) | -0,06 (-0,20 ; 0,10) | |
| Friday | -0,03 (-0,24 ; 0,14) | 0,20 (-0,05 ; 0,22) | -0,05 (-0,21 ; 0,13) | |
| Saturday | 0,09 (-0,25 ; 0,38) | 0,03 (-0,18 ; 0,26) | -0,20 (-0,31 ; 0,14) | |
| Sunday | -0,37 (-0,74 ; 0,06) | 0,16 (-0,18 ; 0,47) | 0,21 (-0,16 ; 0,49) | |
| Work ilr_1_(Standing_work) | -0,07 (-0,24 ; 0,14) | 0,19 (0,05 ; 0,32) | -0,12 (-0,25 ; 0,06) | |
| Work ilr_1_(Standing_work)*Weekday(Monday) | | | |  |
| Tuesday | 0,15 (-0,05 ; 0,39) | -0,202 (-0,352 ; -0,053) | 0,04 (-0,14 ; 0,20) | |
| Wednesday | 0,07 (-0,15 ; 0,26) | -0,113 (-0,262 ; 0,034) | 0,04 (-0,14 ; 0,18) | |
| Thursday | 0,05 (-0,17 ; 0,25) | -0,123 (-0,262 ; 0,034) | 0,06 (-0,09 ; 0,27) | |
| Friday | 0,21 (0,02 ; 0,41) | -0,172 (-0,336 ; -0,03) | -0,05 (-0,24 ; 0,11) | |
| Saturday | 0,18 (-0,18 ; 0,58) | -0,204 (-0,51 ; 0,085) | 0,02 (-0,29 ; 0,32) | |
| Sunday | 0,93 (0,23 ; 1,52) | -0,405 (-0,917 ; 0,089) | -0,51 (-0,98 ; -0,02) | |

*Active=walking, running, stair climbing, and cycling. ilr_1_ = first pivot coordinate, representing the relative importance of a work or leisure time physical behaviour (indicated in parenthesis) with respect to the others. Results based on multivariate multilevel models adjusted for sex, age, smoking-status and BMI. Total number of observations included = 1828. Bold indicates significant at p <0.05, * indicates interaction term.*

**Table A2.** Association between day-to-day leisure time physical behaviours, weekday and relative active work time.

| **Outcome: Leisure composition pivot coordinates** | | | |
| --- | --- | --- | --- |
| **Predictors** | **ilr_1_(Sedentary_Leisure)** | **ilr_1_(Standing_Leisure)** | **ilr_1_(Active_Leisure)** |
|  | β (95 % CI) | β (95 % CI) | β (95 % CI) |
| Weekday (Monday) |  |  |  |
| Tuesday | 0,73 (0,37 ; 1,09) | 0,13 (-0,001 ; 0,29) | 0,006 (-0,13 ; 0,17) |
| Wednesday | -0,15 (-0,33 ; 0,09) | 0,08 (-0,05 ; 0,20) | -0,01 (-0,16 ; 0,12) |
| Thursday | -0,07 (-0,25 ; 0,12) | 0,10 (-0,05 ; 0,23) | -0,05 (-0,18 ; 0,11) |
| Friday | -0,05 (-0,21 ; 0,17) | 0,09 (-0,04 ; 0,23) | -0,05 (-0,24 ; 0,07) |
| Saturday | -0,04 (-0,21 ; 0,17) | 0,03 (-0,23 ; 0,25) | -0,11 (-0,36 ; 0,20) |
| Sunday | 0,09 (-0,20 ; 0,46) | 0,17 (-0,12 ; 0,53) | 0,23 (-0,05 ; 0,56) |
| Work ilr_1_(Active_work) | -0,4 (-0,85 ; 0,03) | -0,22 (-0,36 ; -0,05) | 0,13 (-0,03 ; 0,28) |
| Work ilr_1_(Active_work)*Weekday (Monday) | | | |
| Tuesday | -0,22 (-0,48 ; -0,004) | 0,22 (0,07 ; 0,40) | -0,01 (-0,15 ; 0,14) |
| Wednesday | -0,08 (-0,27 ; 0,14) | 0,13 (-0,06 ; 0,26) | -0,05 (-0,27 ; 0,09) |
| Thursday | -0,06 (-0,28 ; 0,16) | 0,15 (-0,03 ; 0,29) | -0,09 (-0,26 ; 0,08) |
| Friday | -0,18 (-0,39 ; 0,07) | 0,19 (0,04 ; 0,38) | -0,01 (-0,19 ; 0,16) |
| Saturday | -0,11 (-0,66 ; 0,37) | 0,15 (-0,23 ; 0,47) | -0,03 (-0,34 ; 0,28) |
| Sunday | -1,01 (-1,66 ; -0,40) | 0,46 (0,01 ; 1,06) | 0,54 (0,02 ; 0,94) |

*Active=walking, running, stair climbing, and cycling. ilr_1_ = first pivot coordinate, representing the relative importance of a work or leisure time physical behaviour (indicated in parenthesis) with respect to the others. Results based on multivariate multilevel models adjusted for sex, age, smoking-status and BMI. Total number of observations included = 1828. Bold indicates significant at p <0.05, * indicates interaction term.*

**Table A3.** Association between day-to-day leisure time physical behaviours, weekday and relative sedentary work time.

| **Outcome: Leisure composition pivot coordinates** | | | |
| --- | --- | --- | --- |
| **Predictors** | **ilr_1_(Sedentary_Leisure)** | **ilr_1_(Standing_Leisure)** | **ilr_1_(Active_Leisure)** |
|  | β (95 % CI) | β (95 % CI) | β (95 % CI) |
| Weekday (Monday) |  |  |  |
| Tuesday | -0,13 (-0,34 ; 0,10) | 0,14 (-0,03 ; 0,22) | -0,01 (-0,15 ; 0,16) |
| Wednesday | -0,05 (-0,25 ; 0,13) | 0,09 (-0,08 ; 0,25) | -0,03 (-0,18 ; 0,15) |
| Thursday | -0,04 (-0,24 ; 0,14) | 0,11 (-0,06 ; 0,25) | -0,07 (-0,24 ; 0,08) |
| Friday | -0,03 (-0,22 ; 0,17) | 0,10 (-0,09 ; 0,26) | -0,06 (-0,24 ; 0,09) |
| Saturday | 0,07 (-0,24 ; 0,36) | 0,02 (-0,18 ; 0,27) | -0,11 (-0,35 ; 0,11) |
| Sunday | -0,37 (-0,81 ; 0,08) | 0,17 (-0,16 ; 0,52) | 0,23 (-0,14 ; 0,53) |
| Work ilr_1_(Sedentary_work) | -0,02 (-0,12 ; 0,10) | 0,03 (-0,05 ; 0,10) | -0,02 (-0,10 ; 0,05) |
| Work ilr_1_(Sedentary_work)*Weekday (Monday) | | | |
| Tuesday | 0,04 (-0,08 ; 0,16) | -0,03 (-0,11 ; 0,05) | -0,02 (-0,09 ; 0,06) |
| Wednesday | -0,004 (-0,10 ; 0,15) | -0,02 (-0,10 ; 0,05) | 0,02 (-0,05 ; 0,10) |
| Thursday | 0,01 (-0,10 ; 0,12) | -0,03 (-0,12 ; 0,05) | 0,02 (-0,06 ; 0,09) |
| Friday | -0,04 (-0,17 ; 0,08) | -0,01 (-0,20 ; 0,06) | 0,05 (-0,06 ; 0,13) |
| Saturday | -0,08 (-0,29 ; 0,09) | 0,07 (-0,06 ; 0,21) | 0,01 (-0,13 ; 0,16) |
| Sunday | 0,03 (-0,21 ; 0,26) | -0,03 (-0,19 ; 0,14) | -0,003 (-0,14 ; 0,16) |

*Active=walking, running, stair climbing, and cycling. ilr_1_ = first pivot coordinate, representing the relative importance of a work or leisure time physical behaviour (indicated in parenthesis) with respect to the others. Results based on multivariate multilevel models adjusted for sex, age, smoking-status and BMI. Total number of observations included = 1828. Bold indicates significant at p <0.05, * indicates interaction term.*
