# Supplementary material for: Day-to-day pattern of work and leisure time physical behaviours: are low socioeconomic status adults couch potatoes or work warriors?
Source: BMC Public Health. 2021 Jul 7;21:1342. doi: 10.1186/s12889-021-11409-0 (PMC8265073; doi:10.1186/s12889-021-11409-0)
Supplement: Supplementary file 4 — Additional file 4. Assessment of potential selection bias. Comparison of baseline characteristics between blue-collar workers excluded from and included in the study. [file 12889_2021_11409_MOESM4_ESM.docx]

**Additional file 4**

**Table A1**. Baseline characteristics of the blue-collar workers excluded from and included in the study.

|  | **Excluded from study (n=244)** | | | **Included in study (n=963)** | | | P |
| --- | --- | --- | --- | --- | --- | --- | --- |
|  | N(%) | Mean(SD) | Range | N(%) | Mean(SD) | Range |  |
| Age in years | 244(100) | 44.7(10.2) | 19.0;64.0 | 963(100) | 44.9(10.0) | 18.0;68.0 | 0.80 |
| Seniority in years | 230(94) | 11.6(10.2) | 0.1;43.0 | 911(95) | 13.2(10.4) | 0.0;48.0 | 0.03 |
| Overall health (1-5)^A^ | 203(83) | 2.2(0.6) | 1.0;4.0 | 943(98) | 2.3(0.7) | 1.0;5.0 | 0.24 |
| BMI in kg/m2 | 203(83) | 27.3(4.8) | 16.4;40.1 | 947(98) | 27.2(4.9) | 16.2;45.1 | 0.90 |
| Aerobic capacity (ml O2/min/kg) | 147(60) | 30.0(7.0) | 13.0;48.9 | 718(75) | 32.0(9.0) | 13.6;70.8 | 0.01 |
| Alcohol consumption (units/week) | 199(82) | 3.2(4.7) | 0.0;30.0 | 952(99) | 3.4(5.1) | 0.0;40.0 | 0.69 |
| Sex |  |  |  |  |  |  |  |
| Men | 116(48) |  |  | 528(55) |  |  | 0.04 |
| Women | 128(52) |  |  | 435(45) |  |  |  |
| Smoking-status |  |  |  |  |  |  |  |
| Smoker | 91(37) |  |  | 319(33) |  |  | 0.19 |
| Non-smoker | 148(61) |  |  | 630(65) |  |  |  |
| Missing | 5(2) |  |  | 14(2) |  |  |  |
| Shift work |  |  |  |  |  |  |  |
| Fixed day job | 152(62) |  |  | 723(75) |  |  | 0.64 |
| Non-fixed day job | 49(20) |  |  | 214(22) |  |  |  |
| Missing | 43(18) |  |  | 26(3) |  |  |  |
| Working sector |  |  |  |  |  |  |  |
| Cleaning | 66(27) |  |  | 175(18) |  |  | >0.01 |
| Manufacturing | 111(45) |  |  | 569(59) |  |  |  |
| Transportation | 26(11) |  |  | 69(7) |  |  |  |
| Health Service | 5(2) |  |  | 19(2) |  |  |  |
| Assemblers | 2(1) |  |  | 33(3) |  |  |  |
| Construction | 9(4) |  |  | 40(4) |  |  |  |
| Garbage Collectors | 3(1) |  |  | 29(3) |  |  |  |
| Mobile Plant Operators | 7(3) |  |  | 11(1) |  |  |  |
| Other^B^ | 4(2) |  |  | 20(2) |  |  |  |
| Missing | 11(4) |  |  | 0(0) |  |  |  |

*^A^High scores indicate higher self-reported heath. ^B^Includes general office clerks and other elementary workers. P=p-value based on two-sample t-test and Chi-squared test.*
